# Long-term health-related quality of life in patients with advanced esophagogastric cancer receiving first-line systemic therapy

**DOI:** 10.1007/s00520-023-07963-5

**Published:** 2023-08-14

**Authors:** Marieke Pape, Pauline A. J. Vissers, Marije Slingerland, Nadia Haj Mohammad, Peter S. N. van Rossum, Rob H. A. Verhoeven, Hanneke W. M. van Laarhoven, on behalf of the Dutch Upper GI Cancer Group (DUCG)

**Affiliations:** 1 Department of Research & Development, Netherlands Comprehensive Cancer Organisation (IKNL), Utrecht, The Netherlands; 2 Department of Medical Oncology, Amsterdam University Medical Centers, Location University of Amsterdam, Meibergdreef 9, 1105 AZ Amsterdam, The Netherlands; 3 Cancer Treatment and Quality of Life, Cancer Center Amsterdam, Amsterdam, The Netherlands; 4 Department of Surgery, Radboud University Medical Centre, Nijmegen, The Netherlands; 5 Department of Medical Oncology, Leiden University Medical Center, Leiden, The Netherlands; 6 Department of Medical Oncology, University Medical Center Utrecht, Utrecht University, Utrecht, The Netherlands; 7 Department of Radiation Oncology, Amsterdam UMC, Location VUmc, Amsterdam, The Netherlands

**Keywords:** Esophageal cancer, Gastric cancer, Quality of life, Treatment failure

## Abstract

**Purpose:**

To investigate the effect of systemic therapy on health-related quality of life (HRQoL) in patients with advanced esophagogastric cancer in daily clinical practice. This study assessed the HRQoL of patients with esophagogastric cancer during first-line systemic therapy, at disease progression, and after progression in a real-world context.

**Methods:**

Patients with advanced esophagogastric cancer (2014–2021) receiving first-line systemic therapy registered in the Prospective Observational Cohort Study of Oesophageal-gastric cancer (POCOP) were included (*n* = 335). HRQoL was measured with the EORTC QLQ-C30 and QLQ-OG25. Outcomes of mixed-effects models were presented as adjusted mean changes.

**Results:**

Results of the mixed-effect models showed the largest significant improvements during systemic therapy for odynophagia (− 18.9, *p* < 0.001), anxiety (− 18.7, *p* < 0.001), and dysphagia (− 13.8, *p* < 0.001) compared to baseline. After progression, global health status (− 6.3, *p* = 0.002) and cognitive (− 6.2, *p* = 0.001) and social functioning (− 9.7, *p* < 0.001) significantly worsened. At and after progression, physical (− 9.0, *p* < 0.001 and − 8.8, *p* < 0.001) and role functioning (− 15.2, *p* = 0.003 and − 14.7, *p* < 0.001) worsened, respectively. Trouble with taste worsened during systemic therapy (11.5, *p* < 0.001), at progression (12.0, *p* = 0.004), and after progression (15.3, *p* < 0.001).

**Conclusion:**

In general, HRQoL outcomes in patients with advanced esophagogastric cancer improved during first-line therapy. Deterioration in outcomes was mainly observed at and after progression.

**Implications for cancer survivors:**

Identification of HRQoL aspects is important in shared decision-making and to inform patients on the impact of systemic therapy on their HRQoL.

**Supplementary Information:**

The online version contains supplementary material available at 10.1007/s00520-023-07963-5.

## Introduction

Health-related quality of life (HRQoL) is an important outcome for patients with esophagogastric cancer, especially in patients with advanced disease whose prognosis is poor [1, 2]. Up to 40% of patients with advanced esophagogastric cancer receive systemic therapy and survival of these patients in population-based settings is approximately 8 months [3–5]. The intention of palliative systemic therapy is to extend survival, while maintaining or improving quality of life [6, 7].

Available data on HRQoL of patients with esophagogastric cancer mainly originate from the curative setting and from randomized controlled trials in the palliative setting [8–12]. A systematic review of phase II/III randomized clinical trials in esophagogastric cancer showed that in 28 out of the 34 palliative systemic treatment arms, HRQoL remained stable during treatment [7]. However, it is unknown if the stable status changes at progression. A previous study of pooled data from two phase III trials in esophagogastric cancer investigated HRQoL during second-line treatment according to the best overall response and reported that in patients with progressive disease mean scores of all EORTC QLQ-C30 scales, with the exception of diarrhea, worsened after 6 weeks compared to baseline [8].

Participation of patients in randomized clinical trials is limited (< 5%) due to strict inclusion criteria [13]. Additionally, patients in clinical trials usually have a better functional status and less comorbidities compared to all patients in daily practice, which could lead to inferior outcomes in a real-world context [14, 15]. Thus, the impact of systemic therapy on HRQoL could differ for patients in daily practice compared to patients in clinical trials. Therefore, the aim of this study was to assess HRQoL longitudinally in a real-world cohort of patients with advanced esophagogastric cancer during first-line treatment, at disease progression, and after progression.

## Methods

### Study design and data source

Patients with unresectable (cT4b), synchronous or metachronous metastatic esophageal (C15.0–C15.9), gastroesophageal junction/cardia (C16.0), or gastric cancer (C16.1–C16.9) diagnosed between 2014 and 2021 registered in the Netherlands Cancer Registry (NCR) and in the Prospective Observational Cohort Study of Oesophageal-gastric cancer Patients (POCOP) were selected (Supplementary Fig. 1) [16]. For the purpose of this study, only patients who initiated first-line systemic therapy were included.

Clinical data was obtained from the NCR. This registry serves the total Dutch population and is based on notification by the national automated pathology archive. For all patients with unresectable advanced or synchronous metastatic disease diagnosed until 2017 and metachronous metastatic disease until 2016, follow-up information, e.g., duration and failure of first-line, was registered in the second half of 2019, except in two hospitals due to logistic constraints. For patients diagnosed after 2017, information on duration and failure of first-line was registered at initial registration if available (i.e., registration is approximately 1 year after primary diagnosis). Information on vital status was available through the linkage of the NCR with the Dutch Personal Records Database and updated until February 1, 2022.

Patient-reported outcomes measures (PROMs) were available through linkage with POCOP. POCOP is a prospective cohort that contains PROMs of patients with esophageal or gastric cancer [16]. This multi-center cohort study started inclusion in December 2015 and currently approximately 3700 patients from 62 centers are included. Patients filled in the PROMs on paper or electronically (as per patient’s choice) at inclusion and 3, 6, 9, 12, 18, 24, and 36 months thereafter. In general inclusion of patients occurs at primary diagnosis, but inclusion may occur during a follow-up visit.

Patients were included in this study if they completed at least one questionnaire in one of the following time frames: baseline (prior to start of first-line systemic therapy), during first-line (from start first-line systemic therapy until 3 weeks after end of first-line therapy), at progression (from 4 weeks prior to progression until 4 weeks after progression of disease or until start of second-line therapy), and after progression (from 4 weeks after progression or from start of second-line therapy until 6 months after progression) (Supplementary Fig. 2). If the “at progression” interval overlaps with the “during first-line” interval, available questionnaires were included in the “at progression” interval. If the second-line therapy started within 4 weeks after progression (e.g., “at progression”), the available questionnaire was included in the “after progression” interval. Subgroup analyses were performed on patients who did not receive radiotherapy for symptom control or placement of a stent and for patients who received second-line systemic therapy. For the subgroup analyses of patients who received second-line therapy instead of “after progression,” “during second-line” was used (from the start of second-line until the end of second-line therapy or 4 weeks prior to progression on second-line therapy).

### Health-related quality of life

The validated cancer-specific European Organisation of Research and Treatment of Cancer (EORTC) Quality of Life Questionnaire (QLQ)-C30 and tumor-specific esophageal questionnaire (QLQ-OG25) were used in this study [17, 18]. The QLQ-C30 includes 5 functioning scales, 3 symptom scales, 6 single items, and a global health status item [17]. The QLQ-OG25 includes 6 symptom scales and 10 single items [18]. Each item is scored on a 4-point Likert scale, except for global health status which is scored on a 7-point Likert scale. Scores of the QLQ-C30 and QLQ-OG25 were linearly transformed to a score between 0 and 100. Missing data were managed according to the EORTC scoring manual. Higher global health status, functioning, and body image scores indicate a better HRQoL, whereas higher symptom scores indicate more severe symptoms.

### Statistical analysis

Outcomes of EORTC QLQ-C30 and QLQ-OG25 were presented as mean scores (standard deviation [SD]). HRQoL scores were adjusted for clinical characteristics using linear mixed-effects models based on availability in the NCR (sex, performance status, number of comorbidities, number of metastatic sites, radiotherapy for symptom control, or placement of a stent). Outcomes were considered improved or worsened if statistically clinically relevant changes were observed. Interpretation of clinically relevant mean changes (small, medium, or large) over time for the QLQ-C30 subscales was performed based on Cocks et al. [19]. Specific guidelines for interpretation of the QLQ-OG25 subscales were unavailable and clinically relevant changes were interpreted according to general guidelines: small (5 to 10 points), medium (10 to 20 points), and large (> 20 points) [20]. *p* values of < 0.05 were considered statistically significant. All analyses were conducted using SAS® version 9.4 (SAS Institute, Cary, NC, USA).

## Results

### Patient characteristics

This study included 335 patients with unresectable or metastatic esophagogastric cancer who received first-line systemic therapy (Table 1). Besides first-line systemic therapy, 74 of 335 patients received radiotherapy for symptom relieve (22.1%) and 26 out of 335 patients received placement of a stent (7.8%). Two hundred thirty-nine out of 335 patients (71.3%) had first-line treatment failure due to disease progression. One hundred forty-four out of 335 patients (43.0%) received second-line therapy after first-line treatment failure due to disease progression.

**Table 1:** Baseline characteristics at primary diagnosis

	All patients (*n* = 335)
Sex, *n* (%)	
Male	258 (77.0%)
Female	77 (23.0%)
Age	
Median (IQR)	65.0 (59.0–70.0)
Comorbidities, *n* (%)	
0	201 (60.0%)
1	89 (26.6%)
≥ 2	32 (9.6%)
Unknown	13 (3.9%)
Performance status, *n* (%)	
0	156 (46.6%)
1	133 (39.7%)
≥ 2	20 (6.0%)
Unknown	26 (7.8%)
Type of disease, *n* (%)	
Unresectable advanced disease	6 (1.8%)
Synchronous metastatic disease	306 (91.3%)
Metachronous metastatic disease	23 (6.9%)
Tumor location, *n* (%)	
Esophageal	201 (60.0%)
Gastroesophageal junction	68 (20.3%)
Gastric	66 (19.7%)
cT stage at primary diagnosis, *n* (%)	
cT1	1 (0.3%)
cT2	58 (17.3%)
cT3	197 (58.8%)
cT4	30 (9.0%)
cTX	49 (14.6%)
cN stage at primary diagnosis, *n* (%)	
cN0	59 (17.6%)
cN1	90 (26.9%)
cN2	128 (38.2%)
cN3	49 (14.6%)
cNX	9 (2.7%)
Histology, *n* (%)	
Adenocarcinoma	305 (91.0%)
Squamous cell carcinoma	26 (7.8%)
Carcinoma NOS	4 (1.2%)
Tumor differentiation, *n* (%)	
Well/moderate	100 (29.9%)
Poorly/undifferentiated	125 (37.3%)
Unknown	110 (32.8%)
Number of distant metastatic sites, *n* (%)	
0	7 (2.1%)
1	204 (60.9%)
≥ 2	124 (37.0%)
Non-regional lymph nodes metastases, *n* (%)	142 (42.4%)
Lung metastases, *n* (%)	43 (12.8%)
Liver metastases, *n* (%)	162 (48.4%)
Peritoneal metastases, *n* (%)	66 (19.7%)
Bone metastases, *n* (%)	41 (12.2%)
Other metastatic sites, *n* (%)	38 (11.3%)
Radiotherapy for symptoms, *n* (%)	74 (22.1%)
Stent placement, *n* (%)	26 (7.8%)
Type of first-line treatment, *n* (%)	
Monotherapy	8 (2.4%)
Doublet	218 (65.1%)
Triplet	26 (7.8%)
Trastuzumab-containing regimen	78 (23.3%)
Non-trastuzumab targeted regimen	5 (1.5%)
*Pembrolizumab*	1 (0.3%)
*Paclitaxel and ramucirumab*	1 (0.3%)
* Capecitabine, cisplatin, and pembrolizumab*	1 (0.3%)
*5-FU, oxaliplatin and bevacizumab*	2 (0.6%)
Type of second-line treatment, *n* (%)	
No second-line treatment	184 (54.9%)
Paclitaxel and ramucirumab	101 (30.1%)
Taxane monotherapy	16 (4.8%)
Non-taxane monotherapy	6 (1.8%)
Doublet or triplet therapy	10 (3.0%)
Targeted containing regimen	18 (5.4%)

Median overall survival for all patients since the start of first-line treatment was 10.3 months (Supplementary table 1). Among patients with first-line treatment failure due to progression, the median overall survival since the progression of the disease was 4.5 months. The median overall survival since the progression of the disease was 6.9 and 1.4 months for patients who received second-line treatment and who did not receive second-line treatment after progression, respectively.

A baseline questionnaire was available for 164 out of 335 patients (49.0%) and was filled in on average 3 weeks prior to the start of first-line therapy (SD 2.5 weeks). The numbers of questionnaires available were 200 (59.6%), 80 (23.8%), and 110 (32.8%) during first-line therapy, at disease progression, and after progression, respectively. The mean time since the start of first-line therapy to completion of the questionnaire was 7.9 (SD 7.4), 26.3 (SD 15.7), and 37.4 (SD 20.3) weeks for time frames during first-line therapy, at progression, and after progression, respectively.

### Global quality of life and functioning scales

**Fig. 1:**
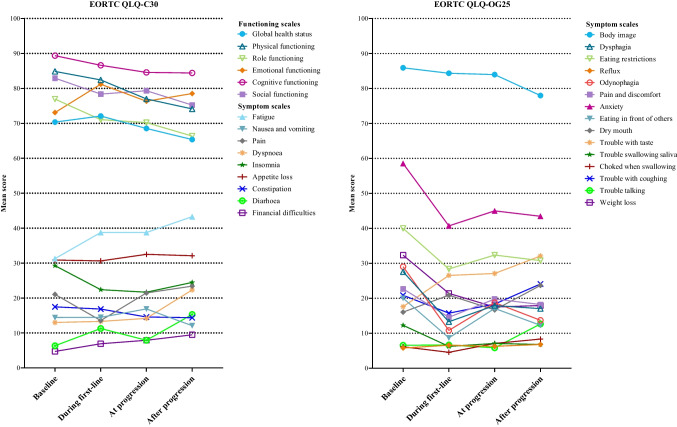
Unadjusted mean scores of the global health status, EORTC QLQ-C30, and QLQ-OG-25 subscales

**Fig. 2:**
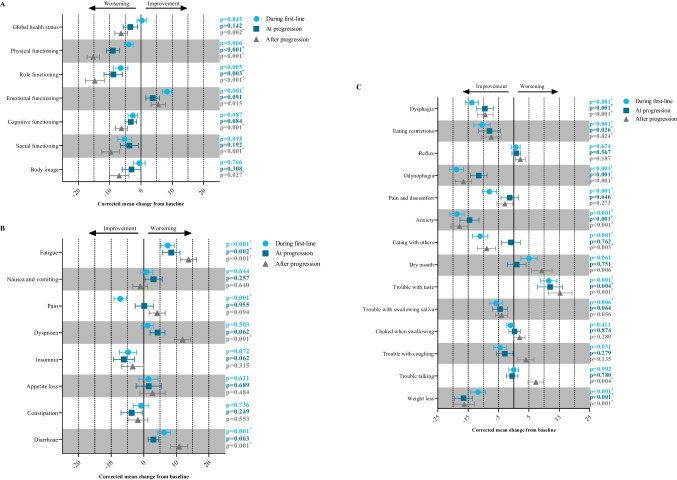
Adjusted mean change from baseline during first-line, at progression, and after progression for outcomes of the EORTC QLQ-C30 functioning scales (**A**), EORTC QLQ-C30 symptom scales (**B**), and EORTC QLQ-OG25 scales (**C**). Clinically significant relevant change according to baseline: ^*^small; ^†^medium; ^§^large

At baseline, mean global health status was 70.3 (unadjusted; Table 2, Fig. 1). Results of the mixed-effect model showed that the global health status remained stable during first-line therapy and at progression, but deteriorated after progression (mean change − 6.3, *p* = 0.002) compared to baseline (Table 3, Fig. 2A). Physical and role functioning remained stable during first-line therapy, but deteriorated at progression (physical: mean change − 9.0, *p* < 0.001; role: mean change − 8.8, *p* = 0.003) and after progression (physical: mean change − 15.2, *p* < 0.001; role: mean change − 15.2, *p* < 0.001) as compared to baseline. Cognitive (mean change − 6.2, *p* = 0.001) and social functioning (mean change − 9.7, *p* < 0.001) deteriorated after progression as compared to baseline. Emotional functioning improved during first-line therapy (mean change 8.3, *p* < 0.001).

**Table 2:** Unadjusted mean scores and standard deviation of the global health status, EORTC QLQ-C30, and OG-25 subscales

	Baseline (*n* = 164)	During first-line (*n* = 200)	At progression (*n* = 80)	After progression (*n* = 110)	*p* value
EORTC QLQ-C30					
Global health status	70.3 (19.7)	72.1 (17.8)	68.5 (20.1)	65.4 (18.6)	0.025^1^
Physical functioning	84.8 (18.8)	82.4 (17.3)	77.0 (21.5)	74.1 (22.1)	< 0.001^1^
Role functioning	76.9 (27.0)	71.0 (25.1)	70.2 (26.6)	66.4 (29.6)	0.013^1^
Emotional functioning	73.1 (21.7)	81.2 (17.2)	76.3 (20.9)	78.5 (20.3)	0.002^1^
Cognitive functioning	89.4 (15.5)	86.6 (19.4)	84.6 (18.5)	84.4 (18.5)	0.093^1^
Social functioning	82.9 (23.3)	78.4 (22.9)	79.3 (22.8)	75.2 (25.3)	0.060^1^
Fatigue	31.3 (23.8)	38.7 (22.4)	38.8 (25.4)	43.3 (24.7)	< 0.001^1^
Nausea and vomiting	14.4 (20.3)	14.5 (17.5)	16.9 (21.1)	12.1 (18.5)	0.400^1^
Pain	21.0 (23.4)	13.6 (19.5)	21.5 (25.7)	23.4 (24.5)	< 0.001^1^
Dyspnea	13.0 (21.1)	13.3 (21.4)	14.2 (23.6)	22.3 (25.7)	0.003^1^
Insomnia	29.3 (29.7)	22.4 (24.2)	21.7 (26.0)	24.5 (27.8)	0.067^1^
Appetite loss	30.9 (32.8)	30.6 (31.4)	32.5 (31.4)	32.1 (32.1)	0.960^1^
Constipation	17.5 (24.6)	16.8 (24.9)	14.6 (25.3)	14.4 (24.6)	0.683^1^
Diarrhea	6.3 (16.4)	11.3 (19.9)	7.9 (15.2)	15.3 (23.4)	0.001^1^
Financial problems	4.7 (16.1)	6.9 (18.5)	7.9 (17.8)	9.5 (19.8)	0.178^1^
EORTC QLQ-OG25					
Body image	85.9 (24.6)	84.3 (23.4)	84.0 (25.0)	78.0 (29.1)	0.075^1^
Dysphagia	27.6 (25.5)	13.3 (19.1)	17.9 (22.5)	17.0 (20.5)	< 0.001^1^
Eating restrictions	40.0 (29.8)	28.4 (26.9)	32.4 (27.1)	30.8 (25.6)	0.001^1^
Reflux	5.8 (14.4)	6.6 (15.4)	6.3 (12.8)	6.8 (14.4)	0.938^1^
Odynophagia	29.0 (27.9)	10.8 (17.7)	18.8 (21.4)	13.7 (18.7)	< 0.001^1^
Pain and discomfort	22.7 (25.3)	14.6 (19.1)	19.8 (24.6)	18.2 (21.3)	0.007^1^
Anxiety	58.5 (29.5)	40.7 (24.7)	45.0 (26.6)	43.5 (25.1)	< 0.001^1^
Eating in front of others	20.0 (30.1)	8.7 (19.9)	17.1 (28.6)	12.3 (22.7)	< 0.001^1^
Dry mouth	16.0 (27.0)	20.9 (24.7)	16.7 (23.1)	23.7 (29.3)	0.073^1^
Trouble with taste	17.6 (29.0)	26.5 (29.2)	27.1 (31.4)	32.1 (33.3)	0.001^1^
Trouble swallowing saliva	12.3 (25.4)	6.2 (15.8)	7.1 (16.5)	6.8 (14.9)	0.016^1^
Choked when swallowing	6.1 (15.8)	4.5 (12.4)	7.1 (15.6)	8.3 (15.9)	0.167^1^
Trouble with coughing	20.9 (24.9)	15.7 (20.6)	18.3 (22.4)	24.1 (24.5)	0.017^1^
Trouble talking	6.5 (16.5)	6.6 (15.3)	5.8 (14.8)	12.7 (24.8)	0.015^1^
Weight loss	32.3 (33.0)	21.4 (26.8)	17.5 (24.3)	17.9 (25.7)	< 0.001^1^

^1^ANOVA *F*-test *p* value

**Table 3:** Adjusted mean scores from linear mixed-effect models for the global health status, EORTC QLQ-C30, and OG25 subscales during first-line, at progression, and after progression. Outcomes are presented as mean (standard error) and were adjusted for sex, performance status, number of comorbidities, number of metastatic sites, and radiotherapy for symptom control (time-dependent) and placement of a stent (time-dependent)

	Baseline (*n* = 164)	During first-line (*n* = 200)	At progression (*n* = 80)	After progression (*n* = 110)	During first-line vs baseline	At progression vs baseline	After progression vs baseline	During first-line vs at progression	During first-line vs after progression	At progression vs after progression
EORTC QLQ-C30										
Global health status	66.38 (3.92)	66.68 (3.80)	62.99 (4.12)	60.06 (3.87)	0.845	0.142	0.002^*^	0.109	< 0.001^*^	0.220
Physical functioning	78.50 (3.93)	74.64 (3.83)	69.51 (4.05)	63.27 (4.03)	0.006	< 0.001^*^	< 0.001^†^	0.006^*^	< 0.001^†^	0.002^*^
Role functioning	67.36 (5.69)	60.88 (5.54)	58.54 (5.81)	52.65 (5.73)	0.005	0.003^*^	< 0.001^†^	0.416	0.006^*^	0.043
Emotional functioning	71.85 (4.17)	80.14 (3.98)	75.61 (4.28)	77.38 (4.09)	< 0.001^*^	0.091	0.015	0.032	0.130	0.391
Cognitive functioning	88.24 (3.81)	85.65 (3.78)	85.02 (3.89)	82.03 (3.84)	0.087	0.084	0.001^*^	0.727	0.065	0.110
Social functioning	79.54 (5.09)	74.32 (4.94)	75.85 (5.18)	69.87 (5.09)	0.015	0.192	< 0.001^*^	0.539	0.090	0.036
Fatigue	39.64 (5.02)	46.98 (4.88)	48.07 (5.16)	53.38 (4.98)	< 0.001^*^	0.002^*^	< 0.001^†^	0.671	0.009^*^	0.054
Nausea and vomiting	21.13 (3.94)	22.02 (3.78)	24.13 (4.07)	20.07 (3.82)	0.644	0.257	0.640	0.347	0.320	0.065
Pain	23.49 (4.77)	16.28 (4.56)	23.65 (4.98)	27.62 (4.72)	< 0.001^*^	0.955	0.094	0.005^*^	< 0.001^†^	0.167
Dyspnea	15.57 (4.73)	16.77 (4.65)	19.83 (4.95)	27.50 (4.85)	0.503	0.062	< 0.001^†^	0.233	< 0.001^*^	0.009^*^
Insomnia	32.45 (5.74)	27.66 (5.46)	26.38 (5.71)	29.09 (5.56)	0.072	0.062	0.315	0.636	0.572	0.325
Appetite loss	41.21 (6.66)	42.67 (6.43)	42.76 (6.71)	43.91 (6.57)	0.621	0.689	0.484	0.977	0.716	0.758
Constipation	30.32 (5.06)	29.51 (4.98)	26.58 (5.32)	28.53 (5.04)	0.736	0.249	0.553	0.359	0.719	0.529
Diarrhea	3.82 (3.46)	10.08 (3.52)	6.79 (3.48)	14.71 (3.75)	0.001^*^	0.063	< 0.001^*^	0.103	0.058	0.002^*^
Financial difficulties^1^	–	–	–	–						
EORTC QLQ-OG25										
Body image	77.29 (5.27)	76.73 (5.07)	74.27 (5.44)	70.35 (5.40)	0.766	0.308	0.027^*^	0.364	0.041^*^	0.262
Dysphagia	27.33 (4.52)	13.55 (4.26)	17.86 (4.62)	17.93 (4.35)	< 0.001^†^	< 0.001^*^	< 0.001^*^	0.110	0.065	0.980
Eating restrictions	45.48 (5.84)	35.03 (5.57)	37.46 (5.86)	38.06 (5.61)	< 0.001^†^	0.026^*^	0.024^*^	0.402	0.268	0.847
Reflux	9.34 (3.03)	9.98 (2.96)	10.16 (2.95)	11.51 (2.99)	0.674	0.567	0.187	0.879	0.315	0.295
Odynophagia	29.90 (4.48)	11.03 (4.09)	18.37 (4.37)	13.35 (4.13)	< 0.001^†^	< 0.001^†^	< 0.001^†^	0.002^*^	0.251	0.043^*^
Pain and discomfort	24.25 (4.67)	16.22 (4.38)	22.93 (4.69)	21.36 (4.47)	< 0.001^*^	0.646	0.273	0.006^*^	0.022^*^	0.537
Anxiety	54.85 (5.55)	36.14 (5.26)	40.30 (5.60)	37.01 (5.30)	< 0.001^†^	< 0.001^†^	< 0.001^†^	0.131	0.708	0.257
Eating with others	27.56 (5.00)	16.49 (4.59)	26.64 (5.02)	18.66 (4.64)	< 0.001^†^	0.762	0.003^*^	< 0.001^†^	0.355	0.004^*^
Dry mouth	15.17 (5.32)	20.19 (5.14)	16.15 (5.36)	24.44 (5.35)	0.061	0.751	0.006^*^	0.159	0.140	0.016^*^
Trouble with taste	21.61 (6.25)	33.13 (6.03)	33.61 (6.46)	36.88 (6.35)	< 0.001^†^	0.004^†^	< 0.001^†^	0.877	0.318	0.419
Trouble with swallowing saliva	14.78 (3.82)	8.92 (3.43)	10.26 (3.62)	10.78 (3.45)	0.006^*^	0.064	0.056	0.471	0.306	0.796
Choked when swallowing	11.60 (3.08)	10.57 (2.92)	11.90 (3.16)	13.56 (3.06)	0.411	0.874	0.280	0.419	0.061	0.377
Trouble with coughing	30.26 (4.82)	25.77 (4.62)	27.35 (4.91)	34.29 (4.78)	0.031	0.279	0.135	0.537	< 0.001^*^	0.022^*^
Trouble talking	11.97 (3.60)	11.95 (3.49)	11.44 (3.62)	19.27 (3.93)	0.992	0.780	0.004^*^	0.758	0.002^*^	0.002^*^
Weight loss	47.21 (5.82)	35.45 (5.47)	30.72 (5.64)	30.93 (5.56)	< 0.001^†^	< 0.001^†^	< 0.001^†^	0.076	0.118	0.942

^1^Unable to calculate due to converge limitation. Clinically significant relevant changes: ^*^small; ^†^medium

### Symptom scales

Mixed-effect models for EORTC QLQ-C30 outcomes showed that fatigue significantly worsened at all 3 time frames compared to baseline with a mean change of 7.3 (*p* < 0.001), 8.4 (*p* = 0.002), and 13.7 (*p* < 0.001) during first-line therapy, at progression, and after progression, respectively (Table 3, Fig. 2B). Pain improved during first-line therapy (mean change − 7.2, *p* < 0.001) compared to baseline. Diarrhea worsened during first-line therapy (mean change 6.3, *p* = 0.001) and after progression (mean change 10.9, *p* < 0.001). Dyspnea worsened after progression (mean change 11.9, *p* < 0.001). All other symptoms remained unchanged over time (Table 3).

Mixed-effect models showed that dysphagia, eating restrictions, odynophagia, anxiety, and weight loss improved during first-line therapy, at progression, and after progression compared to baseline (Table 3; Fig. 2C). During first-line therapy, pain and discomfort (mean change − 8.0, *p* < 0.001) and trouble with swallowing saliva (mean change − 5.9, *p* = 0.006) improved compared to baseline. Eating with others improved during first-line therapy (mean change − 11.1, *p* < 0.001) and after progression (mean change − 8.9, *p* = 0.003) compared to baseline. Trouble with taste worsened during first-line therapy (mean change 11.5, *p* < 0.001), at progression (mean change 12.0, *p* = 0.004), and after progression (mean change 15.3, *p* < 0.001) compared to baseline. Dry mouth (mean change 9.3, *p* = 0.006) and trouble talking (mean change 7.3, *p* = 0.004) worsened after progression compared to baseline. The other disease-specific symptoms remained unchanged over time. Comparison of HRQoL outcomes between time frames during first-line therapy, at progression, and after progression is available in Table 3.

### Quality of life outcomes of patients not receiving radiotherapy for symptom control or stent placement

Mixed-effect models among patients who did not receive radiotherapy for symptom control or placement of a stent after diagnosis (*n* = 243) showed that during first-line therapy several disease-specific symptoms including dysphagia (mean change − 10.4, *p* < 0.001), odynophagia (mean change − 15.5, *p* < 0.001), and pain and discomfort (mean change: − 9.4, *p* = 0.002) improved compared to baseline (Supplementary table 2).

### Quality of life outcomes of patients receiving second-line therapy

For patients who received second-line therapy after progression on first-line therapy (*n* = 144), results of mixed-effect models showed that during second-line therapy global health status, physical functioning, fatigue, dyspnea, financial difficulties, trouble with coughing, and trouble with talking worsened compared to the time frame at progression (Supplementary table 3). Eating with others and weight loss improved during second-line therapy compared to the time point at progression.

## Discussion

Besides survival gain, the intent of systemic therapy is to maintain or improve HRQoL. In this real-world study in patients with unresectable or metastatic esophagogastric cancer receiving first-line systemic therapy, we observed that the majority of HRQoL outcomes were maintained or improved during first-line therapy and at progression, but generally deteriorated after progression, even if patients were treated with second-line systemic therapy.

Our findings in this real-world data cohort are in line with a previous meta-analysis of randomized clinical trials investigating HRQoL during first-line treatment [7]. This meta-analysis reported that global health status remained stable during first-line therapy. In addition, in the meta-analysis improvements of > 10 points were observed in emotional functioning, pain, abdominal pain, appetite loss, eating restrictions, and dysphagia. In our study, we also found an improvement in emotional functioning (8 points), pain (7 points), pain and discomfort (i.e., abdominal pain; 8 points), eating restrictions (10 points), and dysphagia (14 points) during first-line treatment.

In our study severe fatigue was already present at baseline and significantly worsened over time. Many factors (modifiable and non-modifiable) have been identified to affect cancer-related fatigue [21]. Particularly for patients with advanced cancer, earlier intervention (i.e., during systemic therapy) is needed and cognitive behavioral therapy or physical exercise programs during systemic therapy have shown to reduce the severity of cancer-related fatigue [22–25]. Despite existing guidelines for cancer-related fatigue among cancer survivors after treatment, the most common long-term effect among cancer patients remains cancer-related fatigue (68%) [26, 27]. Current care for cancer-related fatigue in clinical practice is possibly insufficient and health care professionals may address cancer-related fatigue more often during consultation and refer patients for interventions for cancer-related fatigue, such as cognitive behavioral therapy [25].

The treatment options for dysphagia include stent placement, short-course radiotherapy, or systemic therapy [28]. If life expectancy is > 3 months, radiotherapy is recommended for palliation of dysphagia [29–31]. In our study, among patients who did not receive radiotherapy or placement of a stent for symptom control, dysphagia, odynophagia, and pain and discomfort improved during first-line therapy, although the improvements were smaller compared to the total population. This may suggest that if immediate relief of tumor-specific symptoms is not needed, the effect of systemic therapy for symptom control could be awaited. Radiotherapy or stent placement could then be used as an intervention when needed later.

In contrast to the time frame during first-line therapy, during second-line therapy no improvements in symptoms were observed compared to the time point at progression, with the exception eating with others and worrying about weight loss which improved. Further deterioration in the quality of life was limited to a few functioning and symptom scales, implying that second-line therapy might be able to stabilize HRQoL.

The main strength of our study is the use of real-world data, which provides a representation of the HRQoL of patients in clinical practice. Furthermore, previous research into the representativeness of patients in POCOP as a reflection of the total esophagogastric cancer population in the Netherlands showed that patients receiving palliative systemic therapy participating in POCOP adequately reflect the total population of patients receiving palliative systemic therapy [32].

Our study also has several limitations. Our results could be biased as not all patients had completed questionnaires at all time periods and patients with poorer functional status or more severe side effects of systemic therapy could be more likely unable to fill in a questionnaire. Additionally, symptom burden differs between patients with esophageal and gastric cancer and for different treatment regimens, however due to limited sample size separate analyses were not performed. For patients diagnosed after 2017, follow-up was limited to approximately 1 year after diagnosis and for patients with long-term response or stable disease after first-line systemic therapy, information on disease progression was unavailable. In patients who did not receive second-line therapy, survival since disease progression was only 1.7 months and the number of patients who filled in a questionnaire after progression (i.e., from 4 weeks after date of progression) was too limited for analysis (*n* = 19).

In conclusion, our study showed that first-line systemic therapy results in the maintenance or improvements of HRQoL in patients with unresectable or metastatic esophagogastric cancer in daily practice. Our results also showed that in patients who did not receive radiotherapy or placement of a stent for symptom control, improvements in symptoms were still observed. In patients who received second-line therapy, the majority of HRQoL remained unchanged, and several outcomes deteriorated. This population-based data on HRQoL adds valuable real-world information to existing evidence from randomized controlled trials that can aid in informing patients, shared decision-making processes, and management of expectations.

## Supplementary Information

Below is the link to the electronic supplementary material.

Supplementary file1 (DOCX 1126 KB)

## Data Availability

The data underlying this article is available at the Netherlands Comprehensive Cancer Organisation (IKNL) upon justified request.
