# Supplementary material for: Long-term health-related quality of life in patients with advanced esophagogastric cancer receiving first-line systemic therapy
Source: Support Care Cancer. 2023 Aug 14;31(9):520. doi: 10.1007/s00520-023-07963-5 (PMC10425291; doi:10.1007/s00520-023-07963-5)
Supplement: Supplementary file 1 — Supplementary file1 (DOCX 1126 KB) [file 520_2023_7963_MOESM1_ESM.docx]

**
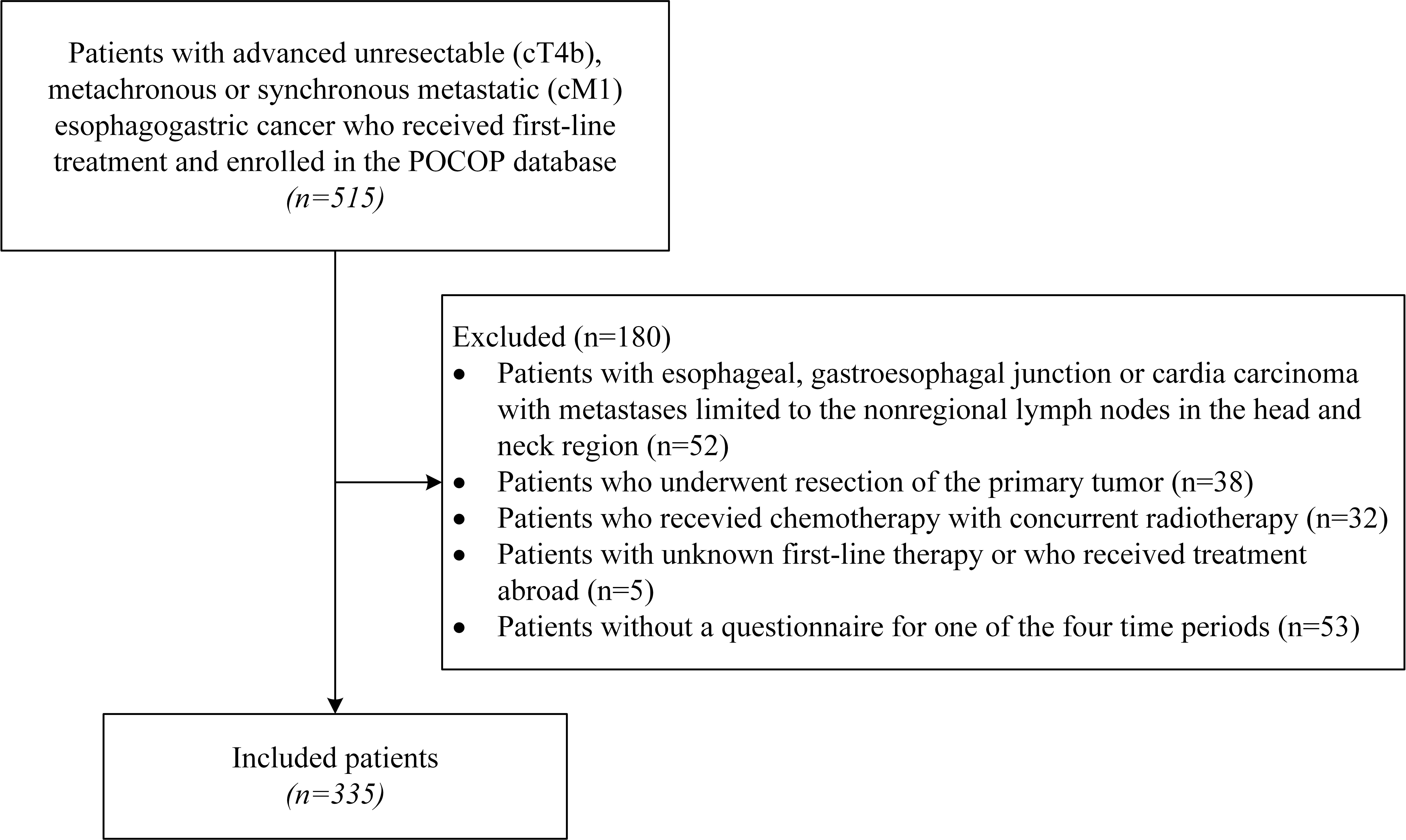
**

**Figure 1. Flowchart of patients inclusion.**


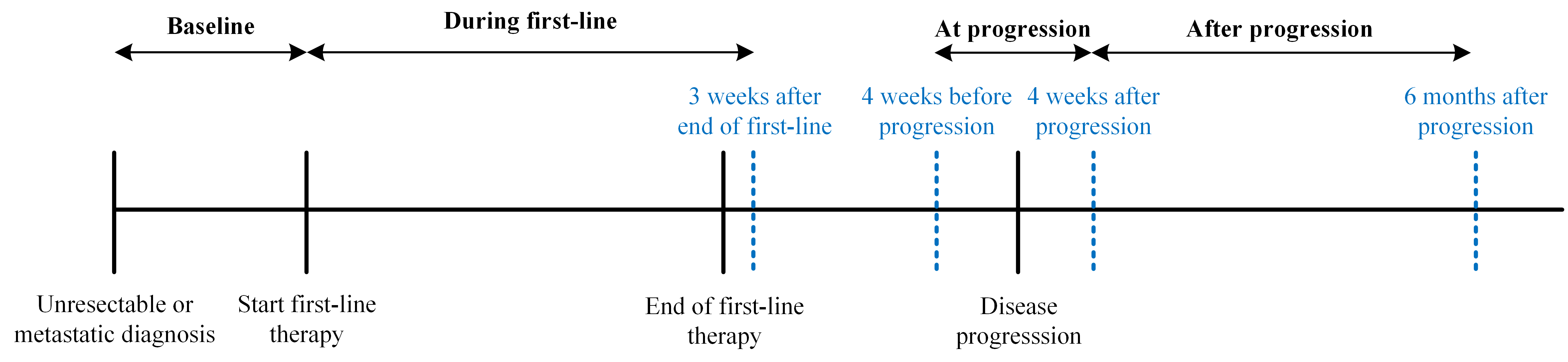


**Figure 2. Schematic over of the inclusion periods.** Definition of the inclusion periods baseline, during first line, at progression and after progression. If the ‘at progression’ interval overlaps with the ‘during first-line’ interval available questionnaires are included in the ‘at progression’ interval. If the second-line therapy started within 4 weeks after progression (e.g. ‘at progression’), the available questionnaire was included in the ‘after progression’ interval.

**Table 1. Overall survival of all patients and patients with first-line treatment failure due to progression of disease.**

| **Starting point of OS analyses** | **Population** | **Number of patients** | **Events** | **Median overall survival (IQR)** (months) |
| --- | --- | --- | --- | --- |
| Start of first-line treatment | All patients | 335 | 270 | 10.3 (5.5-15.2) |
| Progression of disease | Patients with first-line treatment failure due to progression | 239 | 204 | 4.5 (1.8-8.9) |
|  | *Patients receiving second-line treatment after progression* | *144* | *120* | *6.9 (4.2-11.4)* |
|  | *Patients not receiving second-line treatment after progression* | *95* | *84* | *1.4 (0.6-2.6)* |

**Table 2. Adjusted mean scores from linear mixed-effect models for the global health status, EORTC QLQ-C30 and OG25 subscales during first line, at progression and after progression for patients who did not receive radiotherapy for symptom control or placement of a stent.** Outcomes are presented as mean (standard error) adjusted for sex, performance status, number of comorbidities and number of metastatic sites.

|  | **Baseline**  (n=104) | **During first-line**  (n=156) | **At progression**  (n=62) | **After progression**  (n=86) | **During first-line**  **vs**  **Baseline** | **At progression**  **vs**  **Baseline** | **After progression**  **vs**  **Baseline** | **During first-line**  **vs**  **At progression** | **During first-line**  **vs**  **After progression** | **At progression**  **vs**  **After progression** |
| --- | --- | --- | --- | --- | --- | --- | --- | --- | --- | --- |
| **EORTC QLQ-C30** |  |  |  |  |  |  |  |  |  |  |
| Global health status | 66.31 (3.76) | 66.69 (3.71) | 63.44 (4.08) | 59.07 (3.97) | 0.837 | 0.258 | 0.003^*^ | 0.189 | <0.001^*^ | 0.107 |
| Physical functioning | 76.51 (3.54) | 74.13 (3.53) | 67.85 (3.86) | 60.60 (4.02) | 0.149 | <0.001^*^ | <0.001^†^ | 0.003^*^ | <0.001^†^ | 0.003^*^ |
| Role functioning | 67.50 (5.17) | 62.20 (5.14) | 60.84 (5.51) | 52.70 (5.77) | 0.056 | 0.040 | <0.001^†^ | 0.668 | 0.006^*^ | 0.009^*^ |
| Emotional functioning | 66.97 (4.06) | 77.91 (3.95) | 74.05 (4.40) | 75.95 (4.30) | <0.001^†^ | 0.008^*^ | 0.001^*^ | 0.115 | 0.348 | 0.416 |
| Cognitive functioning | 83.58 (3.62) | 82.27 (3.69) | 80.05 (3.85) | 76.94 (3.94) | 0.471 | 0.085 | 0.002^*^ | 0.279 | 0.018^*^ | 0.154 |
| Social functioning | 74.75 (4.95) | 71.23 (4.86) | 72.79 (5.19) | 64.94 (5.42) | 0.180 | 0.536 | 0.008^*^ | 0.575 | 0.043^*^ | 0.013^*^ |
| Fatigue | 38.78 (4.78) | 46.47 (4.70) | 49.22 (5.06) | 54.68 (5.09) | 0.004^*^ | 0.001^†^ | <0.001^§^ | 0.315 | 0.004^*^ | 0.089 |
| Nausea and vomiting | 13.98 (3.78) | 17.64 (3.68) | 20.39 (4.15) | 14.93 (3.92) | 0.129 | 0.044^*^ | 0.716 | 0.272 | 0.219 | 0.032 |
| Pain | 24.99 (4.56) | 17.24 (4.44) | 23.24 (4.83) | 29.52 (4.89) | 0.001^*^ | 0.542 | 0.124 | 0.026^*^ | <0.001^†^ | 0.051 |
| Dyspnoea | 16.19 (4.29) | 16.14 (4.29) | 21.31 (4.71) | 26.47 (4.75) | 0.979 | 0.038^*^ | <0.001^*^ | 0.069 | <0.001^*^ | 0.092 |
| Insomnia | 32.45 (5.40) | 28.29 (5.21) | 27.28 (5.62) | 30.34 (5.67) | 0.182 | 0.164 | 0.594 | 0.737 | 0.480 | 0.327 |
| Appetite loss | 41.96 (6.32) | 44.37 (6.16) | 45.18 (6.67) | 46.53 (6.83) | 0.505 | 0.495 | 0.339 | 0.818 | 0.592 | 0.749 |
| Constipation | 31.58 (4.22) | 35.72 (4.40) | 30.66 (4.96) | 33.73 (4.85) | 0.114 | 0.792 | 0.523 | 0.167 | 0.536 | 0.376 |
| Diarrhoae | 8.82 (3.62) | 14.37 (3.75) | 9.87 (3.67) | 18.91 (4.27) | 0.025^*^ | 0.540 | 0.002^*^ | 0.057 | 0.117 | 0.003^*^ |
| Financial difficulties | 6.04 (3.76) | 4.49 (3.65) | 6.89 (4.02) | 7.92 (4.03) | 0.261 | 0.733 | 0.436 | 0.255 | 0.089 | 0.681 |
| **EORTC QLQ-OG25** |  |  |  |  |  |  |  |  |  |  |
| Body image | 76.47 (5.14) | 78.45 (4.98) | 74.17 (5.61) | 69.67 (5.69) | 0.417 | 0.542 | 0.058 | 0.177 | 0.012 | 0.260 |
| Dysphagia | 29.71 (4.41) | 19.34 (4.22) | 22.66 (4.77) | 23.90 (4.66) | <0.001^†^ | 0.033^*^ | 0.068 | 0.271 | 0.092 | 0.698 |
| Eating restrictions | 41.46 (5.77) | 34.83 (5.51) | 36.19 (6.04) | 39.07 (5.89) | 0.051 | 0.241 | 0.545 | 0.677 | 0.168 | 0.419 |
| Reflux | 10.98 (2.95) | 12.87 (2.96) | 12.90 (2.97) | 13.78 (3.04) | 0.302 | 0.264 | 0.121 | 0.984 | 0.582 | 0.464 |
| Odynophagia | 24.65 (4.50) | 9.17 (4.09) | 14.49 (4.52) | 10.85 (4.36) | <0.001^†^ | 0.005^†^ | <0.001^†^ | 0.039^*^ | 0.449 | 0.189 |
| Pain and discomfort | 26.53 (4.77) | 17.15 (4.49) | 24.03 (4.91) | 22.13 (4.82) | 0.002^*^ | 0.466 | 0.171 | 0.011^*^ | 0.051 | 0.511 |
| Anxiety | 68.12 (5.50) | 43.22 (5.36) | 45.12 (5.81) | 43.52 (5.58) | <0.001^§^ | <0.001^§^ | <0.001^§^ | 0.545 | 0.907 | 0.628 |
| Eating with others | 17.15 (5.02) | 9.98 (4.70) | 18.81 (5.29) | 9.36 (4.96) | 0.019^*^ | 0.636 | 0.024^*^ | 0.006^*^ | 0.804 | 0.002^*^ |
| Dry mouth | 27.07 (5.00) | 31.53 (4.89) | 27.82 (5.11) | 37.82 (5.46) | 0.159 | 0.821 | 0.007 | 0.218 | 0.053 | 0.006 |
| Trouble with taste | 26.42 (5.73) | 38.55 (5.61) | 41.28 (6.33) | 46.18 (6.48) | <0.001^†^ | 0.002^†^ | <0.001^†^ | 0.442 | 0.075 | 0.278 |
| Trouble with swallowing saliva | 11.75 (3.77) | 8.21 (3.58) | 8.16 (3.71) | 8.66 (3.78) | 0.118 | 0.104 | 0.200 | 0.983 | 0.825 | 0.818 |
| Choked when swallowing | 6.21 (2.49) | 5.57 (2.44) | 6.34 (2.84) | 8.44 (2.82) | 0.644 | 0.952 | 0.268 | 0.664 | 0.103 | 0.332 |
| Trouble with coughing | 30.18 (4.28) | 27.63 (4.21) | 26.16 (4.66) | 36.22 (4.77) | 0.271 | 0.195 | 0.058 | 0.604 | 0.003^*^ | 0.004^†^ |
| Trouble talking | 14.89 (3.37) | 14.56 (3.18) | 14.86 (3.39) | 22.70 (4.03) | 0.879 | 0.989 | 0.017^*^ | 0.869 | 0.003^*^ | 0.009^*^ |
| Weight loss | 41.02 (5.65) | 30.50 (5.51) | 25.78 (5.85) | 27.20 (5.87) | <0.001^†^ | <0.001^†^ | <0.001^†^ | 0.130 | 0.300 | 0.676 |

Clinically significant relevant changes: ^*^, small; ^†^, medium; ^§^, large.

**Table 3. Adjusted mean scores from linear mixed-effect models for the global health status, EORTC QLQ-C30 and OG25 subscales during first line, at progression and during second line for patients who received second-line therapy after failure of first-line due to disease progression.** Outcomes are presented as mean (standard error) and were adjusted for sex, performance status, number of comorbidities, number of metastatic sites and radiotherapy for symptom control (time-dependent) and placement of a stent (time-dependent).

|  | **Baseline**  (n=54) | **During first-line**  (n=100) | **At progression**  (n=54) | **During second-line**  (n=55) | **During first-line**  **vs**  **Baseline** | **At progression**  **vs**  **Baseline** | **During second-line**  **vs**  **Baseline** | **During first-line**  **vs**  **At progression** | **During first-line**  **vs**  **During second-line** | **At progression**  **vs**  **During second-line** |
| --- | --- | --- | --- | --- | --- | --- | --- | --- | --- | --- |
| **EORTC QLQ-C30** |  |  |  |  |  |  |  |  |  |  |
| Global health status | 76.13 (6.51) | 74.95 (6.65) | 76.54 (6.65) | 70.98 (6.68) | 0.639 | 0.865 | 0.051 | 0.537 | 0.131 | 0.049^*^ |
| Physical functioning | 81.79 (4.77) | 76.37 (4.82) | 75.52 (4.86) | 70.26 (4.99) | <0.001^*^ | 0.010^*^ | <0.001^†^ | 0.648 | 0.011^*^ | 0.004^*^ |
| Role functioning | 74.83 (8.57) | 71.83 (8.81) | 75.09 (8.70) | 68.99 (8.95) | 0.314 | 0.931 | 0.171 | 0.332 | 0.427 | 0.035 |
| Emotional functioning | 75.32 (5.96) | 85.27 (5.94) | 85.01 (6.08) | 88.13 (6.13) | <0.001^†^ | <0.001^†^ | <0.001^†^ | 0.912 | 0.209 | 0.224 |
| Cognitive functioning | 86.51 (4.82) | 84.16 (5.09) | 83.88 (5.13) | 80.74 (5.17) | 0.240 | 0.240 | 0.020^*^ | 0.896 | 0.151 | 0.126 |
| Social functioning | 86.46 (7.05) | 79.57 (7.37) | 84.01 (7.28) | 84.65 (7.26) | 0.022^*^ | 0.406 | 0.524 | 0.103 | 0.070 | 0.688 |
| Fatigue | 32.57 (7.82) | 39.44 (7.99) | 35.92 (8.12) | 44.57 (8.02) | 0.012^*^ | 0.266 | <0.001^†^ | 0.257 | 0.088 | 0.010^*^ |
| Nausea and vomiting | 15.88 (5.13) | 14.28 (5.14) | 16.05 (5.47) | 12.65 (5.47) | 0.622 | 0.966 | 0.409 | 0.464 | 0.509 | 0.155 |
| Pain | 21.15 (7.22) | 15.39 (7.33) | 14.03 (7.24) | 17.34 (7.37) | 0.020^*^ | 0.002^*^ | 0.171 | 0.504 | 0.450 | 0.136 |
| Dyspnoea | 19.28 (5.93) | 21.57 (6.14) | 20.57 (6.26) | 28.24 (6.43) | 0.347 | 0.514 | 0.006^*^ | 0.726 | 0.042^*^ | 0.022^*^ |
| Insomnia | 27.54 (8.42) | 22.34 (8.56) | 16.36 (8.56) | 17.60 (8.71) | 0.166 | 0.002^†^ | 0.025^†^ | 0.044^*^ | 0.103 | 0.705 |
| Appetite loss | 40.87 (9.94) | 38.79 (10.23) | 37.25 (10.50) | 37.26 (10.63) | 0.656 | 0.528 | 0.540 | 0.667 | 0.748 | 0.998 |
| Constipation | 23.58 (7.63) | 23.55 (8.00) | 19.42 (8.04) | 24.49 (8.36) | 0.994 | 0.321 | 0.852 | 0.264 | 0.806 | 0.204 |
| Diarrhoae | 6.59 (5.48) | 9.53 (5.76) | 5.56 (5.60) | 12.42 (6.16) | 0.341 | 0.577 | 0.136 | 0.145 | 0.374 | 0.056 |
| Financial difficulties | 5.67 (6.06) | 3.04 (6.03) | 4.34 (6.16) | 10.12 (6.28) | 0.129 | 0.605 | 0.043^*^ | 0.525 | 0.002^*^ | 0.041^*^ |
| **EORTC QLQ-OG25** |  |  |  |  |  |  |  |  |  |  |
| Body image | 83.91 (7.79) | 82.36 (7.90) | 80.38 (8.07) | 80.98 (8.39) | 0.503 | 0.199 | 0.519 | 0.423 | 0.723 | 0.887 |
| Dysphagia | 21.11 (5.44) | 8.96 (5.69) | 11.06 (5.95) | 7.92 (5.81) | <0.001^†^ | 0.008^†^ | <0.001^†^ | 0.503 | 0.697 | 0.195 |
| Eating restrictions | 34.10 (8.39) | 24.20 (8.56) | 25.14 (8.81) | 23.39 (8.73) | 0.020^*^ | 0.093 | 0.030^†^ | 0.786 | 0.806 | 0.595 |
| Reflux | 11.65 (4.15) | 12.98 (4.44) | 13.07 (4.34) | 12.28 (4.39) | 0.556 | 0.481 | 0.755 | 0.954 | 0.717 | 0.546 |
| Odynophagia | 21.49 (7.11) | 4.28 (7.05) | 9.58 (7.18) | 3.82 (7.21) | <0.001^†^ | 0.006^†^ | <0.001^†^ | 0.057 | 0.871 | 0.052 |
| Pain and discomfort | 20.49 (7.23) | 11.89 (7.13) | 16.90 (7.29) | 10.66 (7.21) | 0.035^*^ | 0.337 | 0.007^*^ | 0.071 | 0.633 | 0.054 |
| Anxiety | 51.02 (8.34) | 30.53 (8.44) | 31.29 (8.57) | 25.12 (8.52) | <0.001^§^ | <0.001^†^ | <0.001^§^ | 0.797 | 0.037^*^ | 0.069 |
| Eating with others | 25.48 (8.06) | 15.43 (8.10) | 20.84 (8.13) | 14.46 (8.22) | 0.025^†^ | 0.271 | 0.025^†^ | 0.067 | 0.750 | 0.026^*^ |
| Dry mouth | 16.80 (7.86) | 24.92 (8.17) | 20.63 (8.23) | 27.58 (8.34) | 0.021^*^ | 0.318 | 0.009^†^ | 0.190 | 0.466 | 0.068 |
| Trouble with taste | 18.93 (8.85) | 31.60 (8.94) | 33.78 (9.67) | 30.78 (9.46) | <0.001^†^ | 0.017^†^ | 0.026^†^ | 0.603 | 0.868 | 0.570 |
| Trouble with swallowing saliva | 6.57 (4.43) | 1.58 (4.32) | 1.52 (4.42) | 2.58 (4.50) | 0.081 | 0.084 | 0.155 | 0.976 | 0.634 | 0.621 |
| Choked when swallowing | 4.43 (3.63) | 6.04 (3.88) | 6.24 (3.98) | 8.88 (4.03) | 0.377 | 0.372 | 0.046 | 0.914 | 0.168 | 0.195 |
| Trouble with coughing | 29.02 (7.75) | 27.30 (7.92) | 27.75 (8.10) | 36.57 (8.13) | 0.602 | 0.728 | 0.046^*^ | 0.893 | 0.006^*^ | 0.025^*^ |
| Trouble talking | 5.99 (4.11) | 7.30 (4.34) | 7.19 (4.39) | 15.48 (5.14) | 0.552 | 0.614 | 0.008^*^ | 0.947 | 0.011^*^ | 0.011^*^ |
| Weight loss | 36.28 (8.91) | 25.91 (8.89) | 19.16 (8.95) | 12.05 (8.90) | 0.005^†^ | <0.001^†^ | <0.001^§^ | 0.039^*^ | <0.001^†^ | 0.021^*^ |

Clinically significant relevant changes: ^*^, small; ^†^, medium; ^§^, large.
